# Single Nanoparticle Tracking Reveals Efficient Long-Distance Undercurrent Transport in Upper Fluid of Bacterial Swarms

**DOI:** 10.1016/j.isci.2019.11.012

**Published:** 2019-11-08

**Authors:** Jingjing Feng, Zexin Zhang, Xiaodong Wen, Jianfeng Xue, Yan He

**Affiliations:** 1 Department of Chemistry, Key Laboratory of Bioorganic Phosphorus Chemistry & Chemical Biology (Ministry of Education), Tsinghua University, Beijing 100084, China; 2 State and Local Joint Engineering Laboratory for Novel Functional Polymeric Materials, College of Chemistry, Chemical Engineering and Materials Science, Soochow University, Suzhou 215123, China; 3 Centre for Soft Condensed Matter Physics and Interdisciplinary Research, Soochow University, Suzhou 215006, China

**Keywords:** Transport Phenomena, Biological Sciences, Biophysics, Laser Biophysics

## Abstract

Flagellated bacteria move collectively in a swirling pattern on agar surfaces immersed in a thin layer of viscous “swarm fluid,” but the role of this fluid in mediating the cooperation of the bacterial population is not well understood. Herein, we use gold nanorods (AuNRs) as single particle tracers to explore the spatiotemporal structure of the swarm fluid. Individual AuNRs are moving in a plane of ∼2 μm above swarms, traveling for long distances in high speed without interferences from bacterial movements. The particles are lifted and transported by collective mixing of small vortices around bacteria during localized clustering and de-clustering of motile cells. Their motions fit the Lévy walk model, revealing efficient fluidic flows above the swarms. These flows provide obstacle-free highways for long-range material transportations, allow swarming bacteria to perform population-level communications, and imply the essential role of the fluid phase on the emergence of large-scale synergy.

## Highlights

• Active bacterial movements lift single Au nanorods (AuNRs) above the motile cell layer
• The AuNRs are advected by collective hydrodynamic flows induced by swarming bacteria
• They could be transported long distances actively and efficiently in Lévy walk pattern
• The phenomenon suggests fluid-mediated large-scale cooperation in bacterial community

## Graphical Abstract

**Figure:**
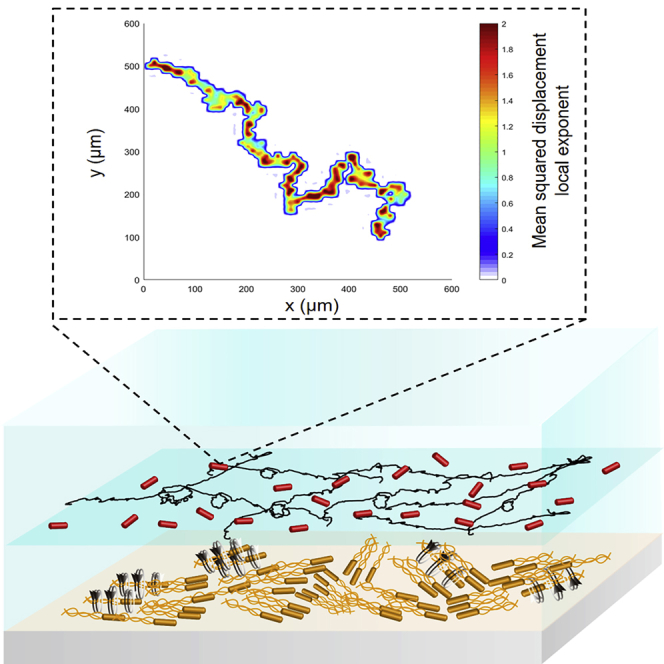

## Introduction

Bacterial swarming is collective migrations of flagellated cells across agar surfaces with swirling patterns (Harshey, 2003, Copeland and Weibel, 2009, Kearns, 2010, Partridge and Harshey, 2013). During swarming, fast-moving bacteria are trapped in a thin layer of viscous fluid called “swarm fluid” (Koch and Subramanian, 2011, Wu and Berg, 2012, Clement et al., 2016, Chuang et al., 2016) The layer of fluid is only micrometers thick and even thinner at the swarm edge and has Reynold number as low as 10^−5^. The swarm fluid can trap surfactants, modify the surface tension of liquids, support the flagella operation, and carry nutrients or other signaling molecules (Kaiser, 2007, Linda et al., 2010, Wu, 2015). A fundamental challenge is to understand the relationships between bacterial cells and the fluid medium (Koch and Subramanian, 2011, Drescher et al., 2011, Mccaskill et al., 2007). Biologically, bacteria can only “sense” changes in the adjacent fluid environment to coordinate their behavior. For instance, in quorum sensing, the cells respond to the accumulations of signaling molecules dispersed in the fluid through gene regulations (Hardman et al., 1998, Miller and Bassler, 2000). The bacteria also react to chemical gradients to control their short-time run lengths through rotating the flagella (Burkart et al., 1998, Wadhams and Armitage, 2004). Theoretical work such as the Vicsek Model (Benjacob, 1995) is based on the assumption of collisions and alignments of a single bacterium with its neighbors in short range. To consider hydrodynamic interactions between motile cells in the context of large-scale collective dynamics (Aditi and Ramaswamy, 2001, Lega and Passot, 2003, Toner et al., 2005, Baskaran and Marchetti, 2009, Gyrya et al., 2010), some physical models treated the swarm fluid as a continuum entangling the bacterial phase and fluid together (Aditi and Ramaswamy, 2001, Gyrya et al., 2010), in which the bacterial community is treated as discrete individual self-propelled particles surrounded by an incompressible and inseparable fluid (Aditi and Ramaswamy, 2001, Wolgemuth, 2008).

Yet experimental evidences based on single particle tracking (SPT) have shown that the fluid environment is complicated and heterogeneous. SPT has long been used as a powerful tool to investigate complex systems (Burov et al., 2011, Metzler et al., 2014). Previous studies have used micro-particles to track the dynamics of bacterial fluid (Grégoire et al., 2001, Patteson et al., 2016). Wu et al. revealed an intensive matter transfer flow (rate v = 8 μm/s) in the leading edge of the swarm using 1 to 2 μm micro-bubbles (Wu and Lubensky, 2011). Zhang et al. found 0.2 μm MgO particles dropped on the liquid-air interface (±20 μm from the swarm front) only diffused normally within a small region (∼4 μm^2^, v = 0.9 μm/s) as the swarm front approaches (Zhang et al., 2010a, Zhang et al., 2010b). Be'Er et al. found 0.5 μm MgO particles were superdiffusive (v = ∼ 9 μm/s) on the upper surface (within 100 μm from the leading edge) (Be'Er and Harshey, 2011). However, since the thickness of the fluid layer is comparable with the dimension of the particles, micron-sized tracers could either have obvious collisions with the motile cell bodies or be trapped at the liquid-air interface, making the tracers' motions incapable of reflecting the real motions of the pure fluid medium.

Here, using *Bacillus subtilis* as a model swarming system (Kearns and Richard, 2010), we introduced 40 × 84 nm gold nanorods (AuNRs) as tracers into the upper region of the swarm fluid near the edge of the swarming bacteria monolayer. Because of their good photostability and low cytotoxicity (Xiao et al., 2010, Xu et al., 2014, Peng et al., 2015), plasmonic AuNRs have been used as tracers for long-duration observation in biological studies with high temporal and spatial resolution. We observed that the nanotracers move continuously in 2D at ∼2 μm above the bacterial layer, advected by the collective vortices resulting from dynamic clustering of bacteria. They could travel rapidly for a total distance of ∼800 μm over the top of hundreds of bacterial cells, while having no direct physical contact with any of them. Their trajectories could be best described by a non-Gaussian superdiffusive model, Lévy walk (LW). Compared with random Brownian motion, LWs could lead to highly efficient long-range transport in complex fluid (Zaburdaev et al., 2015, Chen et al., 2015, Fedotov et al., 2018). Therefore, mediated by the swarm fluid, the highly active bacterial community creates a dynamically well-organized flow transport network above their own bodies, possibly providing a long-range communication avenue for the population. These discoveries may help to understand the information exchange in the bacterial population and the swarm dynamics. This single nanoparticle tracking method could be potentially applied to probe the fluid dynamics and matter transport in other collective systems.

## Results

### Gold Nanorods Are Lifted above the Swarming Bacteria

**Figure 1:**
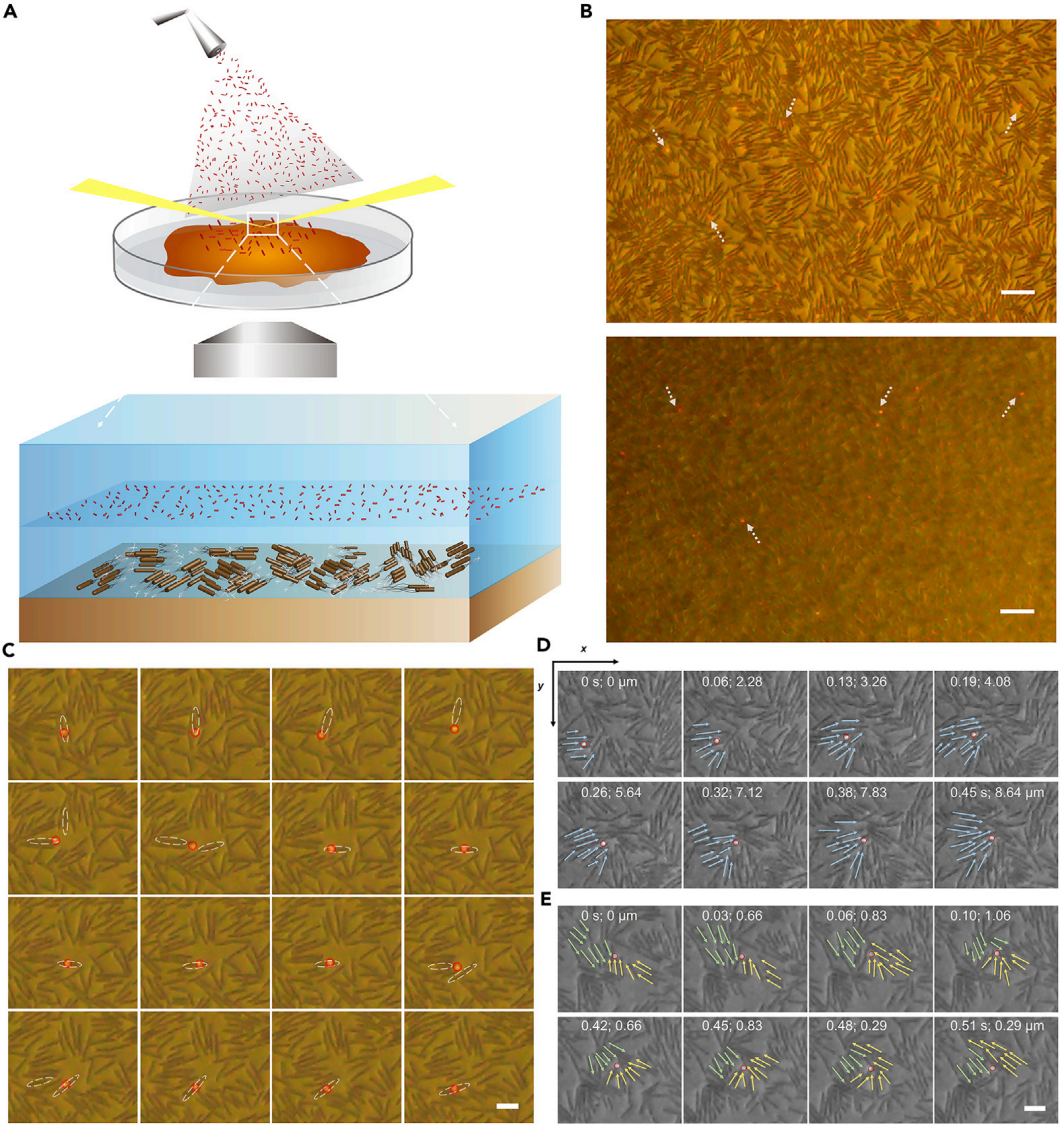
Gold Nanorods (AuNRs) Float above the Swarming Bacteria Layer (A) Schematic diagram of the experimental setup as well as the spatial relationship between the observed AuNR tracers and the swarming bacteria. (B) Typical images with the bacteria (upper) and the AuNRs (lower) in focus, respectively. The single AuNRs are pointed out with a white arrow. The scale bar is 15 μm. (C) A single AuNR crossed over different individual bacterium's body. The white dashed line circles the position of the related bacteria, and the red circles mark the position of the single moving nanorod. The scale bar is 5 μm. (D) An AuNR was transferred in one direction by a “vehicle” consisting of a group of aligned bacteria. (E) The motion of an AuNR slowed down when bacterial clusters encounter “traffic jams” caused by collision. The arrows show the moving direction of the bacteria. The red line circles the nanorod position. The coordinates in (x and y) shows the time (s) and the displacement (μm) of the AuNR. The scale bar is 5 μm. See also Figures S1–S4 and Videos S1, S2, S3, S4, and S5.

To study bacterial swarming, we chose the wild-type *Bacillus subtilis* 3610 strain as the model system (Kearns and Richard, 2010). After ∼2 h inoculation the bacterial colony begins swarming on the wet agar surface and expands outward in swirling patterns, forming a dendrite-like edge (Figure 1A, see the culturing procedures in Transparent Methods). The characteristic scale of the swirls is about 10 μm, and they last for about 0.2 s. To monitor the bacterial fluid by SPT, we used monodispersed 40 × 84 nm gold nanorods (see Figure S1). They were modified with SH-PEG moieties to make them electrically neutral, so as to prevent their adhesion to the negatively charged bacterial cell surface. To introduce the AuNRs into the fluid without causing disturbances, we used a micro-sprayer to atomize the AuNR solution at about 20 mm above the biofilm. By producing an aerosol, the nanotracers naturally fall onto the surfactant fluid surface with minimum interference to the bacterial community. The gravity makes the AuNRs penetrate the liquid rather than float on the air-liquid interface.

For imaging, we used an inverted darkfield microscope with a 20X long working distance objective and a color CMOS camera (see Figure S2). The probing area is near the edge of the monolayer of swarming bacteria where the cells are most active. Under circular oblique illumination, the broadband absorption of the individual bacteria and the plasmonic scattering from single AuNRs allow them to be imaged simultaneously (Figure 1B). The former appears dark and the latter appears red since the plasmonic scattering maximum of the AuNR is 650 nm. This is advantageous over Au nanospheres of similar intensity, which appear yellow-green and can hardly be distinguished from the background color. Interestingly, different from the 3D Brownian motion in a passive homogeneous solution, i.e., being out of focus stochastically, the single AuNRs are seemingly sliding on a 2D plane, with high lateral moving rates but little axial movement (see Video S1). We could generally observe dozens of AuNRs in one frame, and most of the single AuNRs could be tracked in focus continuously for tens of seconds until they run out of the boundaries of the view field. As the particles keep moving in and out, a considerable number of particles can be seen even after 1 h of their addition.

Video S1. Individual AuNRs Move on the Upper Layer of the Swarming Bacteria, Related to Figure 1 The single AuNRs appear as red points. The scale bar is 15 μm.

Close examination of the single AuNR motions indicates that the AuNRs move just like a single bacterium but are relatively independent. They walk, jump, or run stochastically among the bacterial dynamic clusters at fast but varying speeds. At any given time, however, the motion of the single AuNRs could hardly be attributed to the physical contact such as dragging, pushing, or collision from a single bacterium. Although most bacteria appear to be locally clustered together, many AuNR trajectories are straight and elongated (see Video S2) covering several hundred bacteria. Some particles are even able to cross half of the field of view, traveling a net distance of ∼150 μm and a total distance of ∼760 μm. Comparing the sequence of consecutive positions of single AuNRs with nearby single bacteria indicates that the trajectories of nanotracers and cells often cross but do not affect each other (Figure 1C). Therefore, the AuNRs must be transported in a thin 2D layer above the bacterial monolayer. By optical slicing using cross-polarization microscopy, which is a high-resolution confocal-like 3D plasmonic imaging technique based on the polarization properties of anisotropic AuNRs (Cheng et al., 2017), the axial distance between the two layers is determined to be ∼2 μm (see Figures S3 and S4 and the details in the Transparent Methods).

Video S2. Trajectories of the Single Particle Tracking Results of Multiple AuNRs, Related to Figure 1 The scale bar is 10 μm.

Control experiments indicate that the elevation of the single AuNRs results from the dynamic interactions between the nanotracers and the active bacteria. On one hand, not many tracers could exhibit similar behaviors as the 40 × 84 nm AuNRs. Particles of lower density such as 0.5-μm-diameter polystyrene microbeads (see Figure S1, Video S3) stayed afloat on the air/liquid interface and were almost motionless. Heavier particles such as 120 nm Au nanospheres (see Figure S1, Video S4) swiftly penetrated the entire liquid and bacterial layer, sticking to and even sinking into the agar gel. On the other hand, only the swarming bacteria could raise and drive the nanotracers in the fluid. The AuNRs perform Brownian motions both in the culture medium having moving cells but without swarming (diffusion coefficient D = 3.6 μm^2^/s) and in the culture suspensions where the bacteria are filtered out of the medium (D = 2.5 μm^2^/s) (see Figure S5). Moreover, if the bacteria are killed under UV light illumination and stop swarming (see Video S5), the AuNRs that crossed over with bacterial bodies could only undergo slow Brownian diffusion (D = 0.08 μm^2^/s) in the upper viscous solution, and no fast, long-distance transport could be observed (see Figure S5).

Video S3. Polystyrene Microbeads (0.5 μm) Adhere on the Air/Liquid Surface of the Fluid Layer of Swarming Bacteria, Related to Figure 1 The scale bar is 5 μm

Video S4. Gold Nanospheres (120 nm) Sink into the Bottom of the Bacterial Fluid Layer, Rocking and Colliding with the Bacteria Locally in a Small Area, Related to Figure 1 The scale bar is 10 μm

Video S5. AuNRs Move in a Brownian Way in the Fluid Layer after the Bacteria Have Been Exposed to the UV Light Illumination for a Long Period of Time and Stopped Swarming, Related to Figure 1 The scale bar is 5 μm

### The AuNRs Are Advected by the Collective Flow of the Swarms

With no physical contact between the bacterial bodies and the nanotracers, the driving force for the AuNR moving in a 2D fluid layer above the colony can only come from the advection that is generated by the collective fluidic flow from the swirling cell populations. The bacterial swarm fluid has low Reynolds number; hence, the motion of the particles almost completely depends on the local force (Clement et al., 2016). Since the AuNRs appear moving stochastically, the local flow fields must be spatially heterogeneous and change frequently, which can result only from the dynamic clustering of multiple bacteria near the particle. Zhang et al. have reported that bacterial colonies could spontaneously form tightly packed clusters (Zhang et al., 2010a, Zhang et al., 2010b). Within the spatial scale of the clusters, bacterial movements are strongly coordinated, manifesting as a high degree of correlation between cell body orientation, direction of movement, and velocity. Moreover, the dynamic clusters could evolve, collide, fuse, and then re-split into small clusters. Since the axial rotation of a bacterium and its flagella swinging would generate a small vortex around its body, the dynamic clustering of multiple bacteria would inevitably result in collision, fusion, and splitting of their small vortices and provide the forces to drive the AuNRs. Therefore, rather than being carried by individual bacteria, the AuNRs are “hitchhiking” on top of dynamically formed bacterial clusters (Figures 1D and 1E). Notably, these cluster “vehicles” are not stable or long lasting. Whenever encountering “traffic jam,” a cluster will swiftly split and recombine with other individual cells. Meanwhile, the AuNR “passenger” will stop transiently and be transferred to another new “vehicle,” leading to their speed or direction variation. Hence, the transport of the single AuNRs is the result of a synergistic effect of local bacterial vortices and is realized through heterogeneous hydrodynamic flow above the bacterial layer.

**Figure 2:**
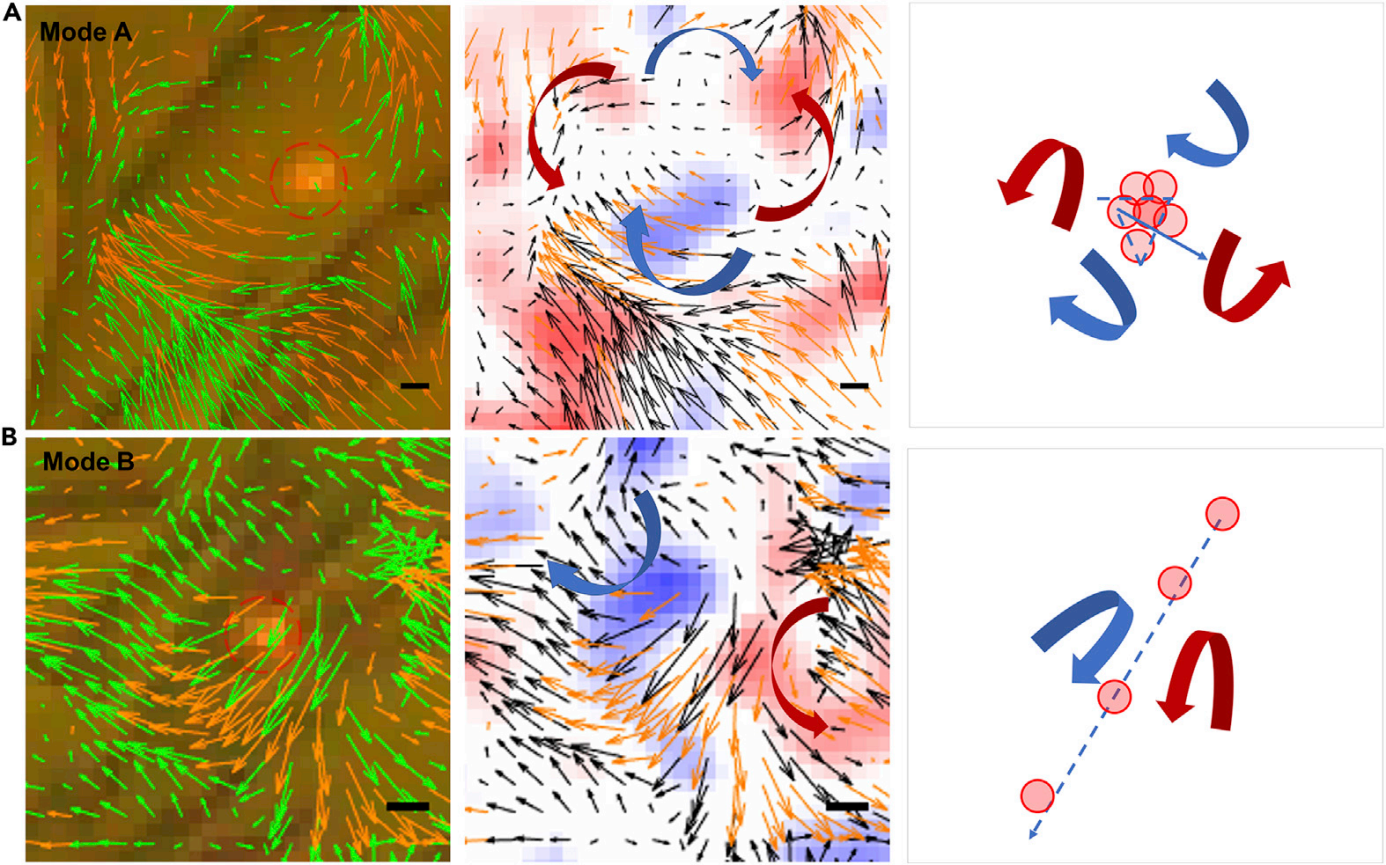
Nanotracer Motion States Are Related to the Local Flow Field Patterns Schematic diagram of (A) the sticking mode and (B) the jetting mode of local flow field patterns. Left panels: the particle image velocity (PIV) vectors overlaid on the raw image. The long dark brown rod is the bacteria and the red-circled spot is the single AuNR. The velocity vectors of PIV are shown in green arrows. The orange arrows are the interpolations. Middle panels: the vorticity color mapping overlaid on the PIV vectors. The velocity vectors of PIV are shown by black and orange arrows. The blue shades refer to the clockwise spin and the red shades refer to the counter clockwise spin. The scale bar is 2.5 μm. Right panels: the red point indicates the position of the particle and the blue line is its trajectory path. In this whole figure, the blue arrow stands for the clockwise flow direction and the red arrow stands for the counter clockwise flow. See also in Transparent Methods and Videos S6 and S7.

Particle image velocity (PIV) analyses (Horn and Schunck, 1980) of the moving bacteria were performed to qualitatively investigate the relationships between the 2D AuNR motions and the swirls created by the swarms. From the local orientation and distribution patterns of the bacteria flow field velocity vectors, we noticed two localized, instant flow field patterns associated with the nanotracers' motions. One is the “sticking mode” (Mode A, Figure 2A), whereby the nanotracers are trapped temporarily or “lingering” in a small area. The magnitude of the velocity vectors of the flow field at the AuNR position is close to 0, resulting from that the rotation of the bacterial swirls around the particle cancel out each other. The other is the “jetting mode” (Mode B, Figure 2B), whereby the nanotracers “jump” large distances in regions between the sticking area. The sum of the flow field velocity vectors and the joint forces of the adjacent bacterial swirls all point to the same direction. Sequential evolution of the velocity vector patterns indicates that the motion of the single AuNRs is alternating rapidly between the two states (see Videos S6 and S7). The “jump-and-linger” mode of the nanotracers appears similar to the “run-and-tumble” mode of the bacterium, making the single AuNRs behave just like active single bacteria (Tailleur and Cates, 2008), although with different underlying mechanisms.

Video S6. Particle Image Velocity (PIV) Vectors Overlaid on the Raw Image, Related to Figure 2 The velocity vectors are shown in green arrows and the interpolations are shown in orange. The yellow circle marks the position of the AuNR being carried across the field. The moving single bacterium appears as long brown rod. The scale bar is 10 μm.

Video S7. PIV Mapping Overlaid on the PIV Vectors, Where the Clockwise Spin Is Shown in Blue Shades and the Counter Clockwise Spin Is Shown in Red Shades, Related to Figure 2 The scale bar is 10 μm.

### The Motions of AuNRs Are Superdiffusive and Non-Gaussian

**Figure 3:**
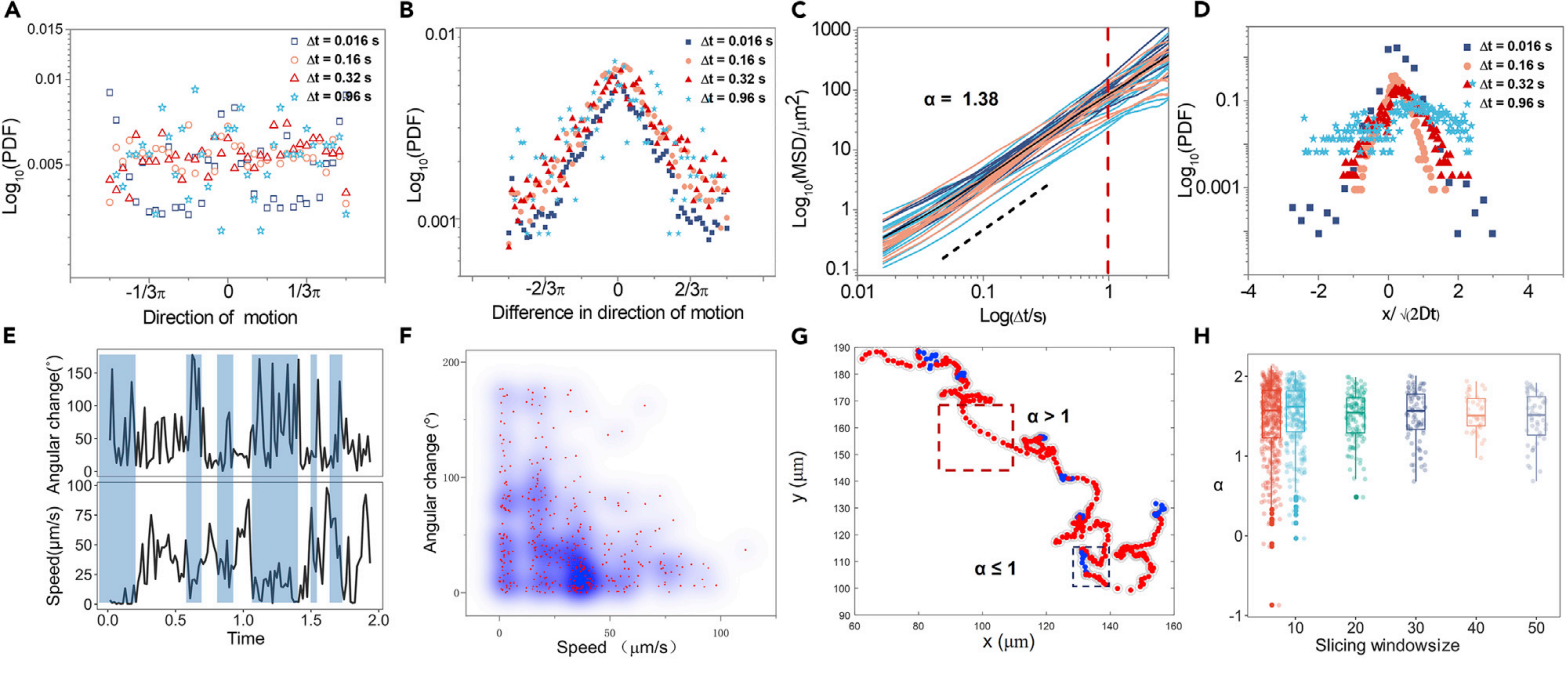
Single Particle Tracking Analysis of the Gold Nanorods (A) Probability density function (PDF) of the direction of motion of single AuNRs with different time intervals (Δ t = 0.016, 0.16, 0.32, 0.96 s). (B) PDF of the difference in motion directions of single AuNRs. (C) Mean squared displacements (MSDs) of multiple single AuNR trajectories. Alpha α = 1.38 shown by black dashed line is the fitted slope of averaged MSD. See also Figure S5. (D) PDF of the scaled displacements of AuNRs. The y axis is in logarithmic scale. Note that the data in (A, B, and D) are calculated by averaging about 57,400 frames. (E) Time-lapsed angular change and the speed of a random sampled trajectory showing the transient motion states between the jump and Brownian-like lingering steps. The rectangles filled with light blue indicate the weak negative correlation between them. (F) The bivariate scattering of the angular change and the speed of the sampled trajectory in (E) with a weak correlation coefficient of −0.33. The blue shades stand for the density. (G) Heatmap of the local scaling parameter α showing the presence of both super-diffusive motion (red, α > 1) and the Brownian motion (blue, α ≤ 1) in a single trajectory. (H) Boxplot added with the scatter of scaling parameter α against the slicing window size.

The phenomenological observation of the AuNR transports in the fluid layer above the bacterial community is further verified through single particle tracking (SPT) analysis. Hundreds of AuNR trajectories are obtained with time duration ranging from 0.02 to 40 s and traveling distances ranging from 0.3 to 800 μm. To get the basic features of the movement, the speed, step size, the motion direction, and the difference in motion direction of the AuNRs are examined. We find that the AuNRs move at a speed about 16 μm/s on average (range from 0.03 to 146 μm/s), and the average step size is about 1.5 μm (0.016 s). As can be seen from Figures 3A and 3B, the single AuNRs at any given time could randomly point to any direction, but the distribution of the variation of the particle directions over a constant time period Δt is centered at zero degree from Δt = 1 to Δt = 60 (0.016–0.96 s), indicating that the particles tend to keep their directions within short time durations. The mean square displacement (MSD) analyses show that the AuNRs are performing superdiffusion with the ensemble-averaged scaling exponent α of 1.38 and a diffusion coefficient of ∼21 μm^2^/s (Figure 3C), nearly 300 times larger than normal diffusion in the pure surfactant liquid. The probability density functions (PDFs) show that the distributions of the scaled displacements at different time intervals deviate from the Gaussian. They are both arrow headed (with a large number of displacements close to zero) and heavy tailed (decaying more slowly than Gaussian at long displacements) (Figure 3D), suggesting a high probability for the AuNRs to move either at very small steps or at large steps.

Careful observations of representative AuNR trajectories indicate that there is a weak negative correlation (with a coefficient of −0.33) between the local speed of the particle and the variation degree of the particle direction (Figures 3E and 3F). The particles tend to keep the original direction when moving fast and change their directions of motion more frequently when the movements slow down, making the trajectory appear to be made up of many large jumps with a lot of short pauses in between. To identify the local regions with different dynamic properties, the instant motion states in the trajectory are revealed by trajectory segmentation using temporal slicing windows. By calculating the local scaling exponent from the MSDs, we found that there exist two states, superdiffusive state (jumps) with *α*>1 and Brownian state (lingering) with *α*≤1 within the same trajectory (Figure 3G). To qualitatively estimate the timescale of the motion states, we tune the size of temporal slicing windows. The sub-trajectories are mostly superdiffusive when the window size is longer than 10 frames. However, when the window size is shortened to 10 frames or less, the local α values exhibit the largest variation (Figure 3H) in the range of *α*≤1, indicating that the Brownian-like movement is more prominent in a time window of less than 0.2 s, which is consistent with the characteristic time of the bacterial swirls.

### Long-Range Transportations of Nanotracers Can Be Described by Lévy Walk

Taken together, from both PIV and SPT analyses, we can develop such a picture: during bacterial swarming, individual bacteria create small vortices around their bodies in the surfactant fluid; the coordination of local vortices creates a spatiotemporally heterogeneous flow field above the bacterial layer, allowing both advected rapid transporting and elevated transient trapping of single nanotracers. Interestingly, although the alternating frequency of the AuNR motion state, or the characteristic time of the bacterial cluster formation and dispersion, is not likely to be greater than ∼0.2 s, the particles have a tendency to keep their walking direction much longer than that. That is, the stochastic switches between the rapid jump and Brownian rest of AuNRs eventually result in their transportation with continually reinforcing directionality.

**Figure 4:**
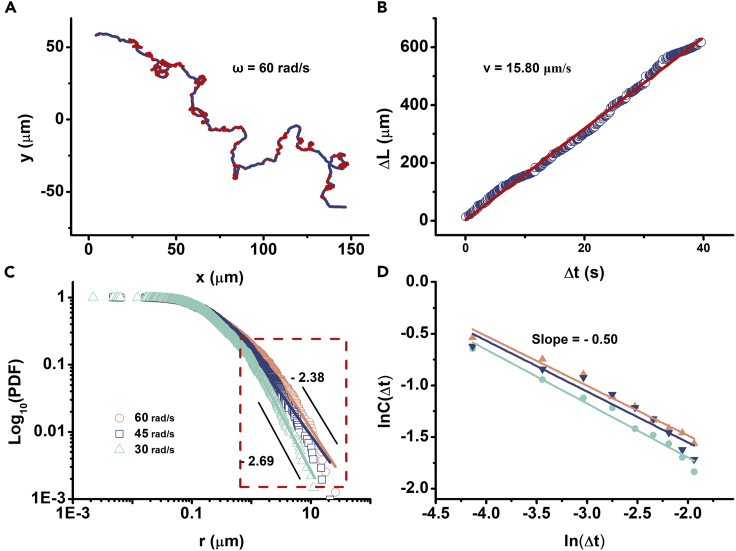
The Motions of AuNRs have Lévy Walk Statistics (A) The typical trajectory of a single AuNR with turning points marked by red with a threshold angle of 60 rad/s. (B) The cumulated distance Δ L of flights as a function of time. The red line is the linear fitting showing a slope of 15.8 μm/s. (C) Log-log plot of the length distributions of flights against distance showing the power law statistics of turn-to-turn flight lengths of the AuNRs. The range of the power law tail is determined by the quality of the fit, highlighted by the red dashed rectangle. The fitted slope of the power-law tail is from −2.38 to −2.69, with different turning angle thresholds (30, 45, and 60 rad/s). (D) The linear fitting of the natural logarithm plot of the power law tail of the velocity autocorrelation function C(t) as a function of Δ t. The color coding stands for different trajectories that collapse well. See details in Transparent Methods.

It has been reported that such directionality persistence, characterized by alternating jump and lingering with superdiffusive and non-Gaussian statistics, often fits with the Lévy process. In particular, the LW model describes that the particles walk large distances at a constant speed with local turnings steps. The walking distances mathematically converge to the power-law distribution with no characteristic scales (Zaburdaev et al., 2015). To test if the single AuNRs were doing LW, “turning points” in trajectories are defined with an instant turning angle larger than a certain threshold (Turchin, 1998) (see details in Transparent Methods in the Supplemental Information). Figure 4A shows a typical long trajectory with a total time of ∼40 s and total travel distance of 760 μm marked with turning points in an angular speed threshold of 60 rad/s. The distances between two consequent turns are considered as a “flight.” We found that the particle moves at a constant speed of ∼16 μm/s through the cumulated length of “flights” as a function of time (Figure 4B). The obtained flight lengths can be fitted with a power law distribution in the tail by using maximum likelihood estimation. In addition, when choosing different turning-angle threshold (30, 45, 60 rad/s), the power law index has a variation from −2.38 to −2.69 (Figure 4C, see Transparent Methods for fitting details). The possibility of matching exponential decays is ruled out by comparing with the Akaike's information criterion weights (Edwards et al., 2007). The power-law model is favored with the AIC weights of 0.99. Another evidence is that the power law tail of the velocity correlation function decays as Δ *t* ^−δ^ with δ = 0.50 in average (Shlesinger et al., 1982, Bouchaud and Georges, 1990, Paeng et al., 2015) (Figure 4D, see Transparent Methods for calculating details). According to the previous study, δ should equal to 2 – α, when α = 1.38 is the scaling superdiffusive exponent calculated from MSD. Therefore, we can conclude that the motion behavior of the AuNRs is consistent with the LW model. It has been reported that the motion of single bacteria in *B*. *subtilis* colony is also LW; however, the statistics is slightly different (Ariel et al., 2015). The superdiffusion exponent of the active bacteria is 1.66, indicating more directionality than the nanotracers. Moreover, to get the power law index value of −2.5, the turning-angle thresholds of the bacteria (5, 10, 15 rad/s) are much smaller than those of the nanotracers, indicating the AuNRs were making turns more dramatically. Therefore, the LW behavior of the AuNRs is similar to the single bacterial motions in the swarm but statistically different.

## Discussion

In bacterial swarming systems, how the multicellular collective patterns emerge from a unicellular structure and how information exchange is performed in a swiftly proliferating and expanding population are still a critical issue to explore. With continuous short-range collisions and alignments, it is hard to imagine the existence of certain special “messenger” bacteria capable of carrying and spreading the information rapidly across a wide space. It must be the viscous fluid environment of the swarms that not only enables the self-propelled bacterial cells to spin their flagella to generate thrusts, but also serves as the medium to carry the nutrients and signals from one location to the other in the colony, e.g., the signaling molecules involved in quorum sensing. A previous study deposited MgO microparticles on the surface of the swarm fluid, and superdiffusive trajectories were obtained (Be'Er and Harshey, 2011). Nevertheless, probably due to the large size, no long-range transportation of the microtracers was observed that was associated with the coordination mechanism at large spatial scales.

**Figure 5:**
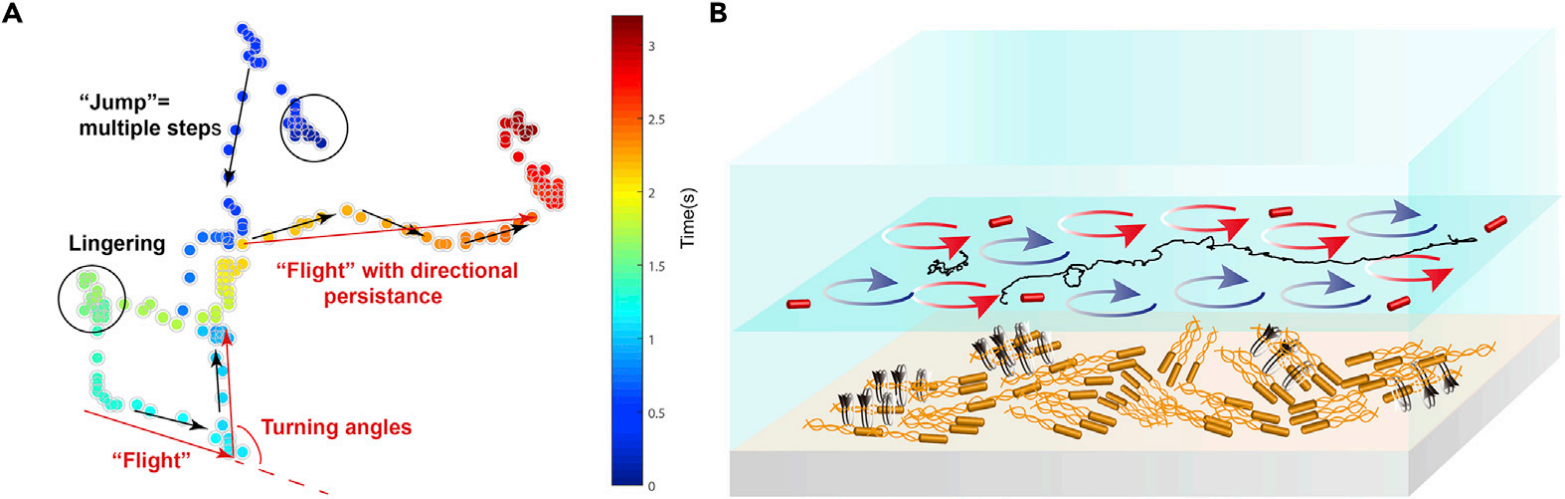
The AuNRs Are Transported in Long Range above the Swarming Bacteria Layer by the Coordination of the Bacteria Vortices (A) Schematic diagram of a representative time-elapsed trajectory of AuNR having jumps, lingering, and Lévy walk “flights” with a directional persistence. (B) Schematic diagram illustrates the long-range transportation of the AuNRs by the cooperation of swarming bacterial vortices. The black arrows stand for vortices around the bacteria. The blue and red circles with arrows stand for the vortices in the fluid layer. The black line draws the trajectory of a single AuNR transported by the living bacterial fluids.

In this work, the nanotracers are far smaller than single bacteria and are lifted above the swarming bacteria. They are neither being pushed by nor “riding” on any single bacterium. Rather, the AuNRs are traveling in 2D in the surfactant fluid layer due to the local synergy of the vortices around the bacteria and exhibit non-Gaussian superdiffusive characteristics with alternating jumps and lingering, consistent with LW statistics (Figures 5A and 5B). Under the Lévy motion mode, the AuNR tracers appear no longer passive and could actively and efficiently travel over long distances across multiple bacterial clusters (Figure 5B). Compared with the LW statistics of individual bacteria showed by Ariel et al. (Ariel et al., 2015, Ariel et al., 2017), the LW of single AuNRs have smaller super-diffusion exponents and larger truncating angular speeds, indicating that the AuNRs are moving more randomly and less directed than the bacteria in the swarms. This could be attributed to the fact that the bacteria are consuming energy and driven by active flagella rotations, but the AuNRs are passively transported by the swarm fluid. Moreover, the bacteria and the AuNRs are spatially divided into two phases with different environments. The single bacteria move in densely packed populations and often encounter physical hindrance from others over short distance. However, the AuNRs travel in an obstacle-free fluid layer above the motile cells. The two similar but different LW transports suggest that the collective movement of high-density bacterial cells produce simultaneously two distinct biological mechanisms. The LWs of the single bacteria may have advantages in foraging or avoiding the hazards, which are associated with continuous adaptation to local environments or even “conflict” with neighbors (Ariel et al., 2015, Ariel et al., 2017). On the other hand, the LWs of AuNR tracers reveal efficient, long-range transportations in the upper fluid medium, which could facilitate communications such as circulations of metabolites and nutrients, fluid mixings for oxygen dissolution, and transports of signaling molecules over long distances. In other words, the two LWs represent the simultaneous near-range competitions and large-scale cooperation between individuals within the same bacterial community, respectively, and the collective motions of all the individuals lead to the emergence of a long-range communication LW network that “links” their activities altogether.

Lévy patterns have been identified in a wide range of organisms, from cells, birds, to even human hunters (Zaburdaev et al., 2015, Edwards et al., 2007, Reynolds, 2009). Most studies consider LW an optimal search strategy being evolved during adaptive interactions with heterogeneous environments. The LW of passive AuNRs, however, is the result of collective local mixing of vortices around the swarming bacterial bodies. Since the vortices have no clear physical boundaries and are short-lived owing to collisions and mixings with each other, our results support a recent hypothesis that the LW can emerge spontaneously and naturally from innocuous responses to the surroundings (Reynolds, 2015). Since individual bacteria are also performing LW, the coincidences and mutual reinforcements of two Lévy patterns apparently lead to higher-level surviving and adaptation to the environment. Similar mechanisms may constitute the underlying physics of the emergence of large-scale coordination in nature such as flocking of birds, schooling of fishes, and collective spreading of cancer cells. Nevertheless, by using nanoparticle tracers, we expect that new collective properties on bacterial and other collective motion systems could be discovered.

### Limitations of the Study

Owing to the limitations of the imaging device, only a small number of AuNRs above swarms could be tracked in long time in our experiments. In future, other optical imaging methods should be developed or applied to achieve high sensitivity and large field-of-view visualization of the material transport network. Furthermore, to fully understand the mechanism behind the obstacle-free highway, physical models or numerical simulations on the motion patterns of the nanotracers based on flow field distribution and dynamic bacteria clustering are required.

## Methods

All methods can be found in the accompanying Transparent Methods supplemental file.

## Supplemental Information

Document S1. Transparent Methods and Figures S1–S5
