# Supplementary material for: Single Nanoparticle Tracking Reveals Efficient Long-Distance Undercurrent Transport in Upper Fluid of Bacterial Swarms
Source: iScience. 2019 Nov 8;22:123–32. doi: 10.1016/j.isci.2019.11.012 (PMC6881698; doi:10.1016/j.isci.2019.11.012)
Supplement: Document S1. Transparent Methods and Figures S1–S5 [file mmc1.pdf]

ISCI, Volume *22*

Supplemental Information

Single Nanoparticle Tracking Reveals Efficient Long-Distance Undercurrent Transport in Upper Fluid of Bacterial Swarms

Jingjing Feng, Zexin Zhang, Xiaodong Wen, Jianfeng Xue, and Yan He

Supplemental Figures

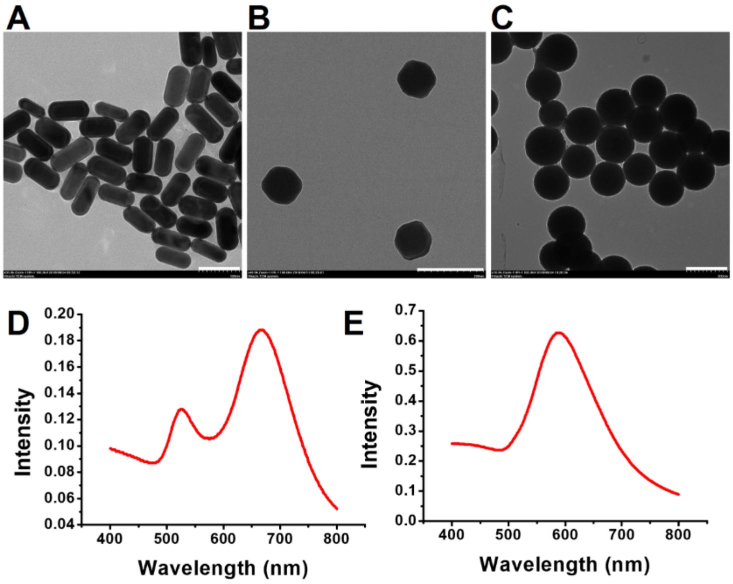

Figure S1. Characterization of the particles used in the experiment. Related to Figure 1A. (A) TEM images of 40×84 nm gold nanorods (AuNRs). The scale bar is 100 nm. (B) TEM images of 120 nm gold nanospheres. The scale bar is 200 nm. (C) TEM images of 0.5 μm polystyrene spheres. The scale bar is 500 nm. (D) UV-vis extinction spectra of the 40×84 nm AuNRs in (A). The longitudinal is 663 nm. (E) UV-vis extinction spectra of the 120 nm gold nanospheres in (B). The peak is 605 nm.

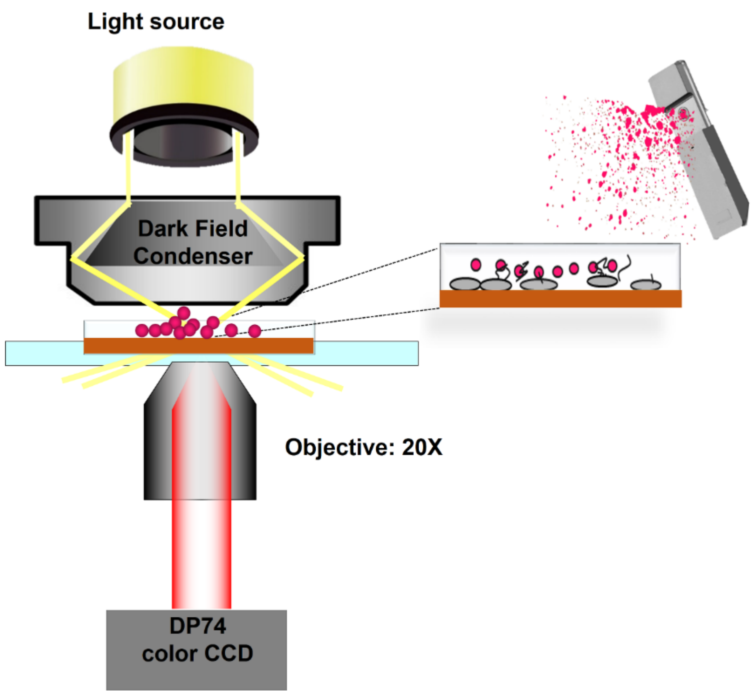

Figure S2. Schematic diagram of the imaging device. Related to Figure 1A. An inverted Nikon Ti-U microscope equipped with a 100 W halogen tungsten lamp, a dark-field air condenser, a 20X long working distance objective lens and an Olympus DP74 color CMOS camera is used to observe the swarming bacteria colony cultured in a 120 mm petri dish. The AuNRs are sprayed into the experimental system.

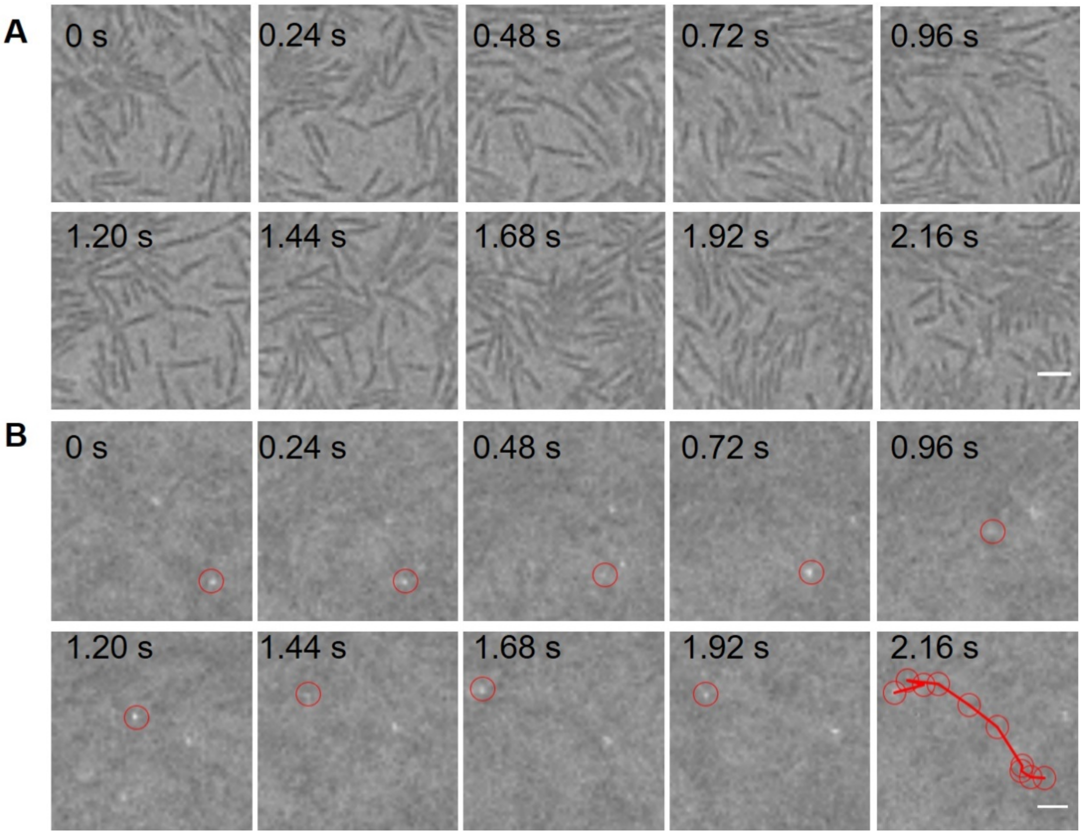

Figure S3. The AuNRs layer and the bacteria layer have different axial position. Related to Figure 1B. (A) Time-lapsed image sequence when the bacteria were in the focal plane. (B) Time-lapsed image sequence when the AuNRs were in the focal plane. The red circle indicates the position of a moving AuNR, and its trajectory is displayed in the last frame. With the aid of a piezo-Z positioning stage, the distance between the layers A and B is determined to be 1.89 μm. The scale bar is 5 μm.

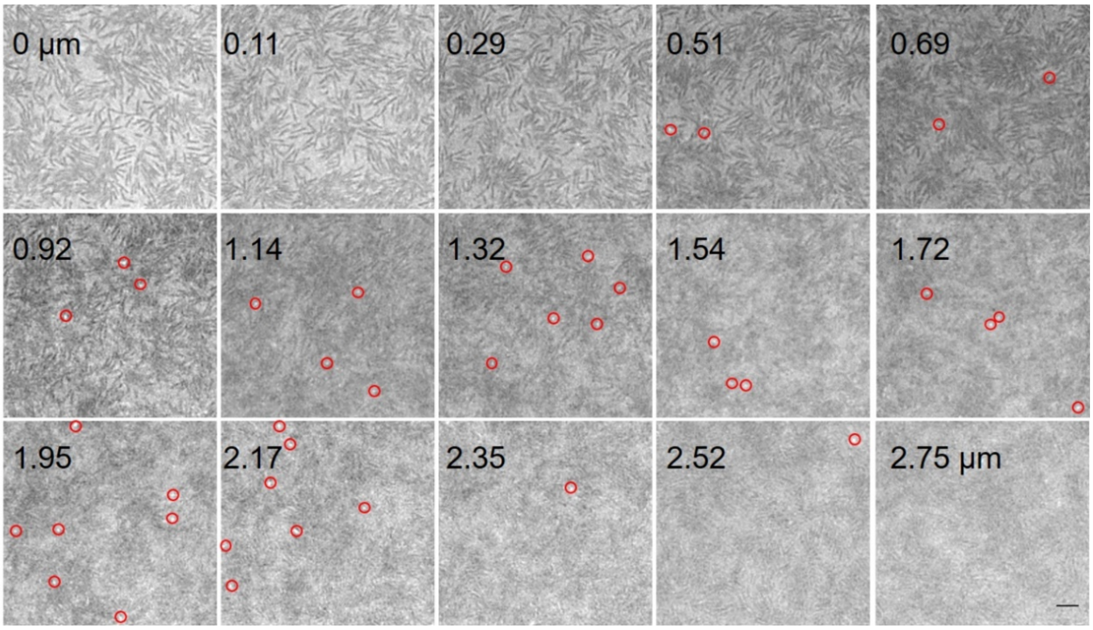

Figure S4. 3D cross polarization imaging of the bacteria and AuNRs. Related to Figure 1B and C. The 3D image sequences are obtained via optical slicing starting from the bacteria layer to the AuNRs layer through manually adjusting the piezo-Z positioning stage. The red circles indicate the single AuNRs. The scale bar is 10 μm.

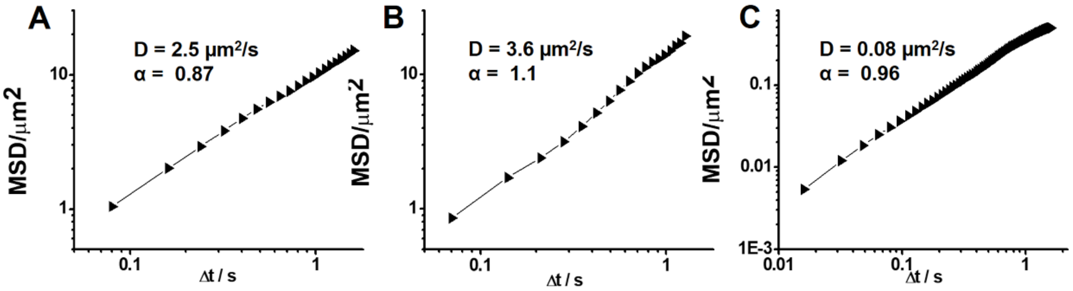

Figure S5. Motion behavior of the single AuNRs above the bacteria layer after the bacteria colony was irradiated under a UV lamp for 6 h. Related to Figure 3C. (A) MSDs of the AuNR movements in the LB cultured fluid medium where the motile bacteria are filtered out with a slope of 0.87 and a diffusion coefficient of 2.5 $\mu m^2/s$. (B) MSDs of the AuNR movements in the LB culturing suspensions where the cells are swimming with a slope of 1.1 and a diffusion coefficient of 3.6 $\mu m^2/s$. (C) MSDs of the AuNR movements in the fluid above the bacteria after the bacteria colony was irradiated under a UV lamp for 6 h with a slope α = 0.96 and a diffusion coefficient of 0.08 $\mu m^2/s$. The MSD curves are calculated by averaging tens of trajectories.

Transparent Methods

Bacteria strain and colony growth

Wild-type *B. subtilis* strain 3610 (purchased from China General Microbiological Culture Collection center) is a gram-positive bacterium with a rod-shaped body and multiple flagella, which generates a propelling force in the direction of its body. They swim with a mean speed about 10 ~ 30 μm/s in a thin liquid film about micrometers thick on the substrate. In our experiments, the bacteria have mean dimensions of 0.7 μm x 4 μm. Colonies were cultured on soft LB agar substrates (10 g/L tryptone, 5 g/L yeast extract, 0.5% agar, all purchased from Bacto Difco, 5 g/L NaCl, purchased from Beijing Chemical Works). For inoculation, 5 μL of *B. subtilis* overnight culture (37 °C, 200 rpm, OD 650 = 0.5) is placed on the center of the agar. The inoculated gel is stored in an incubator at 30 °C. After a lag time of 2 h, the colony starts to expand outward with a speed about 1 cm／hour. After about 2 h, when a colony reached a radius about 2 cm and form 3 - 4 μm thick colonies (Ariel et al., 2015), it could be observed under microscope at the edge of colony where the bacteria almost swarms in a monolayer.

Tracer materials and the micro-spray technology

PEG-modified 40×84 nm gold nanorods were purchased from NanoSeedz and were diluted 20 times before use. Carboxyl modified polystyrene microspheres (0.5 μm) were purchased from Dae technique. Gold nanospheres having size about 120 nm were synthesized using a method reported previously (Zhou et al., 2011). For microspray without disturbing the bacteria colony, we added 50 μL of the chosen particle solution into a high-frequency vibrating atomizer (5W, 5V) and then sprayed it out to a direction parallel to the surface of the colony. The small microdroplets would slowly fall onto the surface of the colony. After that, we immediately took the specimen to the microscope for observation.

Cross polarization microscopy for distance measurements between the AuNRs layer and the bacteria layer

A Nikon LV100ND microscope equipped with a LV-UEPI2 epi illuminator, a 100 W halogen tungsten lamp and an Olympus DP74 color CMOS camera was used for the cross-polarization microscopy measurement. The epi illuminator contains an orthogonal polarizer/analyzer module. We have reported previously that when using single AuNRs as the nanoprobe, background-free confocal-like imaging of the 3D distribution of the AuNRs in biological cells could be obtained via simply adjusting the focusing stage (Cheng et al., 2017). Herein, the images of the AuNRs layer and the bacteria layer were collected using a 20X long working distance objective lens (CFI TU Plan FLUOR BD 20X, NA/WD 0.45/4.5mm), and the distance between the two layers were determined using a piezo-Z positioning stage (PZ-2150). The time-lapsed images were recorded when the AuNRs and the bacteria are in the focal plane respectively. After 3 set of measurements at various positions of different colonies, respectively, the axial distance between the AuNRs layer and bacteria layers is determined to be 2.24 ± 0.16 μm.

Imaging technique and data analysis

The specimen was placed under an inverted microscope (Nikon Ellipse Ti-U) equipped with a 100 W halogen tungsten lamp, a dark-field air condenser, a 20X long working distance objective

lens and an Olympus DP74 color CMOS camera with imaging rate of 60 fps. To localize the precise z-position of the AuNR nanotracer layer relative to the bacteria layer, a confocal microscope equipped with a 20X long working distance objective lens and a 647 nm laser (Nikon A1) was utilized. Single nanoparticle tracking was performed using *Image J* and a code written in *IDL*. Other data analysis was performed using MATLAB and Origin. The segmentation of trajectory using the "angle method" (Turchin, 1998), the quantification of its power-law characteristics, and the evaluation of the power-law model are described in detail in the Transparent Methods. The flow field was measured by using a GUI-based software called PIVlab based on discrete Fourier transform (DFT) (Thielicke and Stamhuis, 2014). The PIVlab software provides high quality flow mapping with powerful boundary value solver interpolation techniques.

The angle method

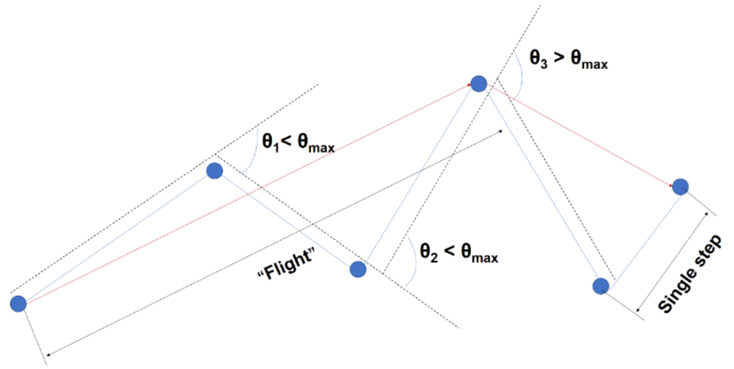

The long trajectory of the AuNR is segmented to several parts by the "angle method" modified from Turchin (Turchin, 1998), which is mainly based on the identification of the reorientation events during single particle walking. As illustrated in the figure above, multiple steps experienced in n time intervals are aggregated into a single "flight" segment, if the turning angle θ between the starting position and the end position is smaller than a predefined angle threshold value θ_{max}. To determine the threshold value θ_{max}, rather than stick to those reported in the literature, we test a wide range of angles from small to large and choose those angles that allow the resulting segment-lengths to converge to the power-law statistics (see discussions below). For the long single AuNR trajectories obtained in this study, it is discovered that whether the θ_{max} is too small (e.g. 5° or 10°) or too large (e.g. >100°), the "flight" segments are either over sampled or under sampled, leading to deviations during the fitting.

Lévy walk models and the power law fitting

To characterize the power law statistics of the Lévy walk model, we used a continuous distribution (Aaron et al., 2009; Ariel et al., 2015),

$$\mathrm{P(x)} = \mathrm{C}\, x^{-\mu}, x \geq x_{min}$$

where α is a constant parameter of the distribution known as the exponent or scaling parameter. The scaling parameter typically lies in the range $1 < \mu \leq 3$, although there are occasional exceptions. Since the distribution diverges at zero, so there must be a lower bound ($x_{min} > 0$) on the power-law behavior. The approach uses log-likelihood fitting methods from the tail started at the lower bound x_{min} that is determined in an automated way by a software developed by Aaron *et al.* (Aaron et al., 2009). For each possible choice of x_{min}, the index

value μ is estimated via the method of maximum likelihood, and the Kolmogorov-Smirnov goodness-of-fit statistic D is calculated. The selected estimation of x_{min} is the value that gives a minimum D over all values of x_{min}. The selection of the power law model over the exponential model is justified using the Akaike weights that calculated the relative likelihoods of either model. See the detailed expression form and calculating methods in the reference article (Edwards et al., 2015)

The auto-correlation function of velocity for LWs

Let $\mathrm{v(t)}$ denotes the velocity at time t of a particle following a LW. In the original LW model (Shlesinger et al., 1982), particles move at constant speed between random reorientations. We defined the velocity auto-correlation function (Paeng et al., 2015) as

$$C(t) = \frac{\sum_t v(t) \cdot v(t' + t)}{\sum_t v(t) \cdot v(t)}$$

where $\langle \cdot \rangle$ denotes averaging over all times t in independent samples of infinite trajectories. Assuming waiting times between reorientation events have a density $\psi(\tau)$ with a power-law tail, $\psi(\tau) \sim \tau^{-\beta+1}$, then we have $\mathrm{C}(\Delta \mathrm{t}) \sim t^{-(\beta-1)}$ (Shlesinger et al., 1982; Bouchaud and Georges, 1990). Here, α=1.38, $\beta = 3 - \alpha$ =1.62 (β is the Levy stable parameter with $\alpha + \beta = 3$). Thus, the C(t) should have a tail with about 0.6. We note that because of the “jump-and-linger” two-state motion of the AuNRs, it is normal to have a small deviation.

Data and Software Availability

All the experimental data and related software will be provided upon request to the authors.

Supplemental References

Aaron C., Shalizi C. R., and Newman, M. E. J. (2009). Power-Law Distributions in Empirical Data. SIAM Rev. 51, 661-703.

Thielicke, W., Stamhuis, E. J. (2014). PIVlab – Towards user-friendly, affordable and accurate digital particle image velocimetry in MATLAB. J. Open Resea. Soft. 2, e30.

Zhou, R., Xiong, B., He, Y. and Yeung, E. S. (2011). Slowed diffusion of single nanoparticles in the extracellular microenvironment of living cells revealed by darkfield microscopy. Anal. Bioanal. Chem. 399, 353-359.
